# A Novel Jumbo Phage PhiMa05 Inhibits Harmful *Microcystis* sp.

**DOI:** 10.3389/fmicb.2021.660351

**Published:** 2021-04-20

**Authors:** Ampapan Naknaen, Oramas Suttinun, Komwit Surachat, Eakalak Khan, Rattanaruji Pomwised

**Affiliations:** ^1^Environmental Assessment and Technology for Hazardous Waste Management Research Center, Faculty of Environmental Management, Prince of Songkla University, Hat Yai, Thailand; ^2^Center of Excellence on Hazardous Substance Management (HSM), Bangkok, Thailand; ^3^Division of Computational Science, Faculty of Science, Prince of Songkla University, Hat Yai, Thailand; ^4^Molecular Evolution and Computational Biology Research Unit, Prince of Songkla University, Hat Yai, Thailand; ^5^Department of Civil and Environmental Engineering and Construction, University of Nevada, Las Vegas, United States; ^6^Division of Biological Science, Faculty of Science, Prince of Songkla University, Hat Yai, Thailand

**Keywords:** *Microcystis*, jumbo phage, cyanophage, aggregation, genome analysis, auxiliary metabolic genes, efficiency of phage killing (EOK)

## Abstract

*Microcystis* poses a concern because of its potential contribution to eutrophication and production of microcystins (MCs). Phage treatment has been proposed as a novel biocontrol method for *Microcystis*. Here, we isolated a lytic cyanophage named PhiMa05 with high efficiency against MCs-producing *Microcystis* strains. Its burst size was large, with approximately 127 phage particles/infected cell, a short latent period (1 day), and high stability to broad salinity, pH and temperature ranges. The PhiMa05 structure was composed of an icosahedral capsid (100 nm) and tail (120 nm), suggesting that the PhiMa05 belongs to the *Myoviridae* family. PhiMa05 inhibited both planktonic and aggregated forms of *Microcystis* in a concentration-dependent manner. The lysis of *Microcystis* resulted in a significant reduction of total MCs compared to the uninfected cells. A genome analysis revealed that PhiMa05 is a double-stranded DNA virus with a 273,876 bp genome, considered a jumbo phage. Out of 254 predicted open reading frames (ORFs), only 54 ORFs were assigned as putative functional proteins. These putative proteins are associated with DNA metabolisms, structural proteins, host lysis and auxiliary metabolic genes (AMGs), while no lysogenic, toxin and antibiotic resistance genes were observed in the genome. The AMGs harbored in the phage genome are known to be involved in energy metabolism [photosynthesis and tricarboxylic acid cycle (TCA)] and nucleotide biosynthesis genes. Their functions suggested boosting and redirecting host metabolism during viral infection. Comparative genome analysis with other phages in the database indicated that PhiMa05 is unique. Our study highlights the characteristics and genome analysis of a novel jumbo phage, PhiMa05. PhiMa05 is a potential phage for controlling *Microcystis* bloom and minimizing MC occurrence.

## Introduction

Blooms of toxic cyanobacteria are an increasing phenomenon in water bodies worldwide. *Microcystis*, the most predominant genus in cyanobloom, has been considered the leading producer of microcystins (MCs) (Harke et al., 2016). MCs have been intensively studied among cyanotoxins due to their toxicity, causing severe liver and kidney damages, tumor promotion, and gastroenteritis (Falconer et al., 1983; Carmichael, 2001; Li et al., 2017). Accumulation of MCs in aquatic ecosystems increases public health concerns (Pham and Utsumi, 2018; Bi et al., 2019).

*Microcystis* is a unicellular microorganism and exhibits high phenotypic plasticity (Xiao et al., 2018). It forms large aggregated colonies in natural freshwater, involving extracellular polymeric substances (EPS). EPS not only regulate buoyancy but also provide protective functions against predator grazing and chemical stressors (Chen et al., 2019). Such characteristics promote global distribution and dominance of *Microcystis*.

Several approaches have been developed to prevent and control cyanobloom, such as nutrient management, artificial mixing and chemical control (Huisman et al., 2018). However, these strategies have faced several problems, including the persistence of toxic residues, time requirement and cost. Biological control using bacteriophage against the cyanobacteria is an attractive idea. Cyanobacterial viruses or cyanophages are abundant during peak-cyanobloom and play an important role in mediating host communities in the ecosystem (Yoshida-Takashima et al., 2012). Cyanobloom can be controlled through the lysis-inducing mortality by phages.

Phages exhibit diverse sizes, morphology, and genome. The vast majority of phages have smaller genomes at tens of kb, while phages with larger genomes are either jumbo phages (>200 kb) (Yuan and Gao, 2017) or megaphages (>500 kb) (Devoto et al., 2019). To date, more than 150 available genome databases of jumbo phages have been reported and exhibited various genomic features. Jumbo phages have been isolated from various environments and infected mostly gram-negative bacteria (Imam et al., 2019).

Genome sequences of jumbo phages are highly divergent. Besides structural components and genome replication proteins, jumbo phages harbor numerous hypothetical proteins and auxiliary metabolic genes (AMGs) that are absent in small phage genomes. The presence of AMGs is implicated in the interception and redirection of host metabolism (Thompson A.W. et al., 2011; Yuan and Gao, 2017). Multisubunit RNA polymerase (RNAP) encoded by jumbo phages cause independence of their replication from the host transcription (Lavysh et al., 2016; Yuan and Gao, 2017). Jumbo phages also harbor elongation factors to maintain the overall translation efficiency during infection (Al-Shayeb et al., 2020). In addition, translation termination elements in jumbo phage rescue ribosomes stalled on damaged transcripts and enhance the degradation of aberrant proteins (Al-Shayeb et al., 2020). Furthermore, AMGs in cyanophage genomes appear to contribute to photosynthesis and pentose phosphate pathways that increase dNTP synthesis for phage replication and supply substantial energy and carbon for phage production (Thompson L.R. et al., 2011; Zimmerman et al., 2020).

To date, eight cyanophages specific to the genus *Microcystis* have been reported (Jaskulska and Mankiewicz-Boczek, 2020). Only three cyanophage genomes (MaMV-DC, Ma-LMM01 and Mic1) have been sequenced and characterized, exhibiting genomes with less than 200 kb (Yoshida et al., 2008; Ou et al., 2015; Yang et al., 2020). All of them carry AMGs responsible for maintaining cyanophage genes and releases of their progeny. Genome comparative analyses indicated that they are phylogenetically distant from other bacteriophages and each other. Based on morphology, MaMV-DC and Ma-LMM01 are grouped in the family *Myoviridae*, while Mic1 belongs to *Siphoviridae*.

Microcystins-producing *Microcystis* is harmful to humans and animals. Many strategies have been used to prevent or reduce *Microcystis* blooms (Huisman et al., 2018). These methods have been successful; however, success is not guaranteed. The purpose of this study was to offer an alternative method using cyanophage as a bio-control against cyanotoxins-producing bacteria to facilitate water management. We isolated and evaluated a cyanophage against MCs-producing *Microcystis*. For the first time, infection kinetics and lysis-mediated MCs release on both planktonic and aggregated cells were investigated. Comprehensive biological characterization and genome analysis of cyanophage were performed. We also examined cyanophage properties, including the virion stability, growth kinetic and viral yield host range.

## Materials and Methods

### Cyanophage Isolation and Plaque Formation

Cyanophages infecting *Microcystis* were isolated using a modified liquid bioassay as described by Chénard and Chan (2017). The *Microcystis* SG03 was previously isolated from the Songkhla Lake (Songkhla, Thailand) and used as the host. The host culture at exponential state (approximately 10^6^ CFU/ml), was prepared in BG-11 broth (pH 7.5) and incubated under 25 μmol photon m^–2^/s (cool fluorescent light), 12 light and 12 h dark cycle at 25°C for 5 days (Belcher and Swale, 1982). Hospital wastewater samples were collected from an aeration basin of activated sludge treatment in January 2020 from Songklanagarind Hospital, Songkhla Province, Thailand. Ten milliliters of the wastewater samples were centrifuged and the supernatant was filtered (0.22 μm pore-size, polyethersulfone membrane, Sartorius, United Kingdom). Five milliliters of the filtrates were added to six-well plates containing 1 ml of the host prepared as mentioned above, while sterile water was used as a negative control. Samples were incubated for up to 10 days under the same conditions described above. Clearance of the host culture indicated the activity of lytic phages. The cultures from the clear wells were collected and centrifuged at 8,000 × *g* for 2 min. The supernatants containing phage (phage lysates) were diluted (10^–4^–10^–20^) with TM buffer (50 mM Tris–HCl and 10 mM MgSO_4_⋅7H_2_O, pH 7.8). An aliquot of 20 μl of the diluted phage lysates was mixed with 180 μl of the host culture in a 96-well plate and incubated under the same conditions described above. Clearance of the host culture was observed daily, and phage lysates were collected from the wells that were clear. This procedure was conducted for five rounds to obtain homogenous phage stock.

The plaque formation was observed using a conventional double-layer agar method. One-hundred microliter of serially diluted cyanophage lysate with TM buffer was mixed with 1 ml of the 10^6^ CFU/ml host culture. Three milliliters of melted top agar containing 0.5% low melted agarose (Bio-Rad) in BG-11 medium were added to the mixture and then overlaid onto a 1% BG-11 agar six-well plate (Thermo Scientific, United States), and incubated under the previously described conditions for 15 days. Homogenous plaques were observed daily. The host culture without cyanophage served as a negative control.

### Cyanophage Purification and Amplification

A conventional cesium chloride (CsCl) step gradient was performed for cyanophage purification (Nasukawa et al., 2017). Twenty milliliters of the exponential host culture were inoculated with 20 ml of cyanophage lysate and incubated under the previously described conditions for 7 days. The mixture was centrifuged and filtered (0.45 μm pore-size, cellulose acetate filters, Sartorius, United Kingdom). The filtrate was precipitated with polyethylene glycol 6,000 (10%, wt/v) and sodium chloride (2M), followed by incubation for 24 h at 4°C. After centrifugation at 10,000 × *g* for 30 min, the pellet was resuspended in 2 ml of TM buffer. The CsCl step gradient was performed by orderly layering CsCl at ρ = 1.0, 0.82, and 0.66 from the tube’s bottom. The cyanophage suspension was slowly layered on top of the gradient tube, and the tubes were loaded in a SW40Ti swing-out rotor (Beckman Coulter, Inc.). After centrifugation at 30,000 × *g*, 4°C for 3 h, a single visible band was collected and dialyzed with TM buffer 4°C for 3 h.

The cyanophage was propagated using a liquid medium method. Briefly, the exponential host culture (450 ml, 10^6^ CFU/ml) was mixed with 50 ml of the purified cyanophage and subsequently incubated for 7 days under the previously described conditions. The mixture was centrifuged and filtered (0.45-μm pore-size, cellulose acetate filters). The cyanophage titers were determined using the conventional double-layer agar method, as described above. The phage lysate was kept at 4°C for further experiments.

### Transmission Electron Microscopy

The cyanophage size and morphology were determined by negatively stained images. Cyanophage lysate was absorbed onto copper grids, stained with 2% uranium acetate, and observed at 80 kV using a JEOL, JEM-2010 transmission electron microscope.

### Host Range Test and Efficiency of Phage Killing

The lytic activity of cyanophage was determined against 10 strains of MCs-producing *Microcystis* isolated from the Songkhla Lake. *Microcystis* culture was prepared as described above. One-hundred microliters of cyanophage (10^8^ PFU/ml) were added to 96-well plates (Thermo Scientific, United States), containing 100 μl of *Microcystis* cultures (10^8^ CFU/ml) (multiple of infectivity: MOI 1). The cultures without cyanophage served as negative controls. Plates were incubated under the conditions described above and observed daily for *Microcystis* cell lysis. The cultures without lysis after 14 days were considered as insusceptible hosts (Ou et al., 2013).

The efficiency of phage killing (EOK) was assessed and classified by modifying the efficiency of plating (EOP) described by Mirzaei and Nilsson (2015). In the EOK, the phage titer was estimated by the Most Probable Number (MPN) (infectious units/ml). Then, the MPN number was used in EOK instead of the plaque forming unit (PFU/ml) in the EOP. The MPN assay was performed using serial dilutions (Suttle and Chan, 1993; Jarvis et al., 2010; Chénard and Chan, 2017). The infectious phage unit was obtained by MPN interpretation. Briefly, cyanophages were serially diluted (10^–4^–10^–20^) with BG-11 in 96-well plates to obtain a final volume of 100 μl solution. One hundred microliters of cyanobacterial cultures (10^6^ CFU/ml) were added into each well and incubated for 7 days under the conditions described above. The numbers and the dilution levels of clear wells were recorded and interpreted through an MPN calculation program (Jarvis et al., 2010), resulting in infectious phage unit. Three independent experiments were conducted. The average infectious phage unit (A) was determined and used for the EOK. The EOK was calculated as:

(1) $$\mathrm{Efficiency}\mathrm{of}\mathrm{phage}\mathrm{killing}\left(\text{EOK}\right)=\frac{At}{Ah}$$

where *At* and *Ah* are the average infectious/killing units on target bacteria and a total number of infectious phage units on the bacterial host, respectively. The EOK values were classified as highly productive (≥0.5), medium productive (0.1 ≤ EOK < 0.5), low productive (0.001 < EOK < 0.1) or inefficient (≤0.001) (Mirzaei and Nilsson, 2015).

### Clonal Relatedness of *Microcystis* and Measurement of MCs-Production

Clonal relatedness among the 10 *Microcystis* sp. was investigated by the highly iterated palindromic (HIP) PCR method described elsewhere (Wilson et al., 2005). DNA of all isolates was extracted using a NucleoSpin^®^Soil kit (MACHEREY-NAGEL, Germany). The HIP-targeted primer was HIP-CA (GCGATCGCGCA). PCR amplification was performed with a volume of 20 μl containing 5 μl buffer (dNTP, Mg^2+^) (bioline, United Kingdom), 3 μl of primer (1 μM), 0.25 μl MyTaq polymerase (1 unit), 1 μl DNA template (20 ng/ml), and 7.75 μl deionized water. The PCR thermal cycling program included initial denaturation at 95°C for 7 min, followed by 30 cycles at 90°C for 1 min, annealing at 52°C for 1 min, and extension at 70°C for 1 min and a final extension at 70°C for 10 min. PCR products were observed by electrophoresis on 1% agarose gel in 0.5 TBE buffer [89 mM Tris (pH 7.6), 89 mM boric acid, 2 mM EDTA] at 60 volts for 3 h. The 1 kb DNA ladder (Solis BioDyne) was run in parallel. Bionumerics version 7.6 was used to construct a dendrogram.

Intracellular MCs (IMCs) and/or extracellular MCs (EMCs) from *Microcystis* cultures were measured. Briefly, eighty milliliters of *Microcystis* (10^6^ CFU/ml) were cultured as previously described for 5 days. After centrifugation at 10,000 × *g* for 15 min, the supernatant and pellets were collected. The IMCs and EMCs were determined using the pellets and supernatant, respectively. To determine IMCs content, the pellets were suspended in 4 ml of methanol-water solution (50%, v/v) (Dai et al., 2009) and sonicated for 10 min followed by incubation at 50°C for 20 min (Gkelis et al., 2015). The extracted toxins were filtered (0.22 μm pore-size polyethersulfone membrane, Sartorius, United Kingdom). The filtered IMCs and the supernatant containing EMCs were measured using a MCs-ADDA ELISA kit (Product No. 520011, Abraxis, United States) following the manufacturer’s instructions. The measurements were duplicated.

### One-Step Growth Curve

A one-step growth experiment was conducted to evaluate the latent period, burst time and burst size of cyanophage (Ou et al., 2013). Ten milliliters of the cyanophage (10^5^ PFU/ml) were added to 90 mL of the log-phase host culture (10^6^ cell/ml), and the mixture was incubated for 7 days at the conditions described above. The cyanophage titers were determined daily using the conventional double-layer agar method as described above. Three independent experiments were conducted. The latent period was taken as the time interval between viral inoculation and the beginning of the cyanophage production. The burst size was calculated as the number of liberated phage particles minus the unadsorbed cyanophage particles divided by the number of initial bacteria (Kropinski, 2018).

### Phage Stability Under Environmental Stress

Salinity, pH and temperature stability tests were performed. To test phage viability under salinity stress, phage suspensions in TM buffer (pH 7.5) containing various sodium chloride concentrations (0.5, 5, 10, 20, 30, and 40 ppt) were investigated. The different pH values in TM buffer adjusted by 5M HCL and 5M NaOH were obtained to achieve pH 5, 7, 9, and 11. The phage in TM buffer (pH 7.5) was used as a control for salinity and pH stability tests. The phage viability in TM buffer (pH 7.5) was tested at 4, 25, 35, and 45°C. One microliter of the cyanophage (10^5^ PFU/ml) was added to each solution (900 μl) and then incubated at 25°C for 12 and 24 h. Titers of survival cyanophage from each test were estimated using the MPN assay as described above. The experiments were triplicated.

### Planktonic Cell Killing Assay

*Microcystis* killing by cyanophage was conducted in 96-well plates. One-hundred microliters of the *Microcystis* SG03 culture (10^6^ CFU/ml) were added to different cyanophage concentrations to have the multiplicity of infection (MOI) of 0.001, 0.01, 0.1, 1, 10, and 100. The plates were incubated for 7 days under the previously described conditions. Bacterial density was measured daily using optical densitometry (LUMIstar^®^ Omega, Germany) at 580 nm (Myers et al., 2013). The cyanophage titer was estimated on day 7 by the MPN assay as described above. The highest phage titer provided by specific MOIs indicates the optimal ratio between phage particles and host cells (the optimal MOI) (Gašić et al., 2011).

Microcystins concentrations during cyanophage infection were determined. Different cyanophage concentrations were mixed with 20 ml of the host culture (10^6^ CFU/ml) to achieve MOI of 0.01, 1, and 100. After 3- and 5-day incubation periods, the mixtures were centrifuged at 10,000 × *g* for 5 min. The IMCs accumulated in *Microcystis* and lysis-mediated MCs release (LM-MCs) were measured using the cell pellet and supernatant, respectively, as described above. MCs were determined using an ELISA kit as described above. The experiments were conducted in triplicate.

### Aggregated Cell Killing Assay

The ability of cyanophage killing aggregated *Microcystis* cells was evaluated through cell density, cell-bound exopolymers, and MCs. Aggregated cell formation was prepared in 6-well plates (Thermo Scientific, United States), by mixing 5 ml of *Microcystis* culture (5.8 × 10^8^ CFU/ml) with CaCl_2_ (a final concentration of 60 mM) and followed by incubation under the conditions described above for 2 days (Dervaux et al., 2015; Drugǎ et al., 2019). After the aggregation was observed, the medium and planktonic cells were carefully removed, leaving aggregated cells at the bottom of the wells. Cyanophage solutions in BG-11 medium were added into each well to achieve the MOI of 0.1, 1, 10, and 100. The cultures were incubated for 7 days, as described above. Planktonic cell culture and aggregated cells without cyanophage served as controls. The bacterial densities were measured daily using optical densitometry (LUMIstar^®^ Omega, Germany) at 580 nm.

To estimate the amount of cell-bound exopolymers, 200 μl of samples from each well were collected, centrifuged (at 10,000 × *g* for 1 min) and resuspended in 200 μl PBS buffer (137 mM NaCl, 2.7 mM KCl, 10 mM Na_2_HPO_4_, and 1.8 mM KH_2_PO_4_). Two microliters of Alcian blue solution (1%, w/v) in acetic acid (3%, v/v) (pH 2.5) were added and incubated with shaking at 80 rpm for 20 min at 25°C. The remaining Alcian blue in the solution was measured spectrophotometrically at 606 nm, compared to non-aggregated cells without cyanophage. The reduction of Alcian blue dye indicated its adsorption on bacterial EPS (Vandevivere and Kirchman, 1993).

To determine IMCs and LM-MCs during cyanophage infection of aggregated cells at the MOI of 100, 20 ml of the host culture (10^8^ CFU/ml) were mixed with cyanophage (10^10^ PFU/ml) and incubated. After 3 and 7 days of incubation, the cultures were centrifuged at 10,000 × *g* for 15 min. The IMCs accumulated in *Microcystis* and LM-MCs were measured using the cell pellet and supernatant, respectively, as described above. All the experiments were conducted in triplicate.

### Scanning Electron Microscopy

The aggregated cells were inoculated with the phage at the MOI of 100. After 5 days of incubation, 20 ml of the cultures were collected, centrifuged, and the pellet was washed with PBS three times and fixed with 1 ml of 2.5% glutaraldehyde in 0.1 M PBS, pH 7.4, followed by an incubation at 25°C for 2 h. The pellets were dehydrated by a series of ice-cold ethanol (30, 50, 70, 80, 90, and 100%, respectively and 10 min for each concentration). The samples were air dried and coated by gold particles, followed by observation under a scanning electron microscope (Quanta 400, Thermo Fisher Scientific).

### Genome Extraction, DNA Sequencing and Analysis

Cyanophage genome was analyzed as described elsewhere (Higuera et al., 2013). A cyanophage lysate was concentrated through Amicon^®^ Ultra-15 (Sigma, Germany). Two-hundred microliters of concentrated cyanophage were then treated with DNase I and RNase A (Thermo Scientific, United States) according to the manufacturer’s instructions to remove host DNA and RNA. Then, three-hundred microliters of the cyanophage were mixed with 100 μl lysis buffer (1M Tris, pH 8.0, 0.5M EDTA, 10 % SDS and 10 mg/ml proteinase K) and incubated at 60°C for 1 h. An equal volume (approximately 400 μl) of the phenol:chloroform:isoamyl alcohol solution (24:24:1) was added to separate phage DNA. After centrifugation (10,000 × *g*, 10 min), two separate phases were formed. The upper phase solution containing phage DNA was collected. The DNA was precipitated using 0.3 volume of 3 M NaOA and 1 volume of isopropanol followed by incubation at −20°C for 2 h. The mixture was centrifuged (10,000 × *g*, 10 min), and then the DNA pellet was dissolved in 50 μl of sterile distilled water. The DNA quality was checked using electrophoresis (sharp single DNA band) and a NanoDrop spectrophotometer (*A*_260_/*A*_280_ = 1.8–1.9) (MaestroGen Inc.) and kept at −20°C until use.

The whole-genome sequencing of cyanophage was performed with paired-end 150-bp reads on Illumina NovaSeq 6000 (Novogene Co., Ltd., Singapore). The reads quality was investigated using FASTQC (Brown et al., 2017) and trimmed with Trimmomatic 0.39 (Bolger et al., 2014). Resulting reads were *de novo* assembled into contigs using Spades 3.11.1 (Bankevich et al., 2012). The open reading frames (ORFs) were identified using GeneMarkS (Besemer et al., 2001) and PHASTER (Zhou et al., 2011). The annotation of ORFs was performed using Blastp (E-value cutoff = 10^–3^) of NCBI server (Chénard et al., 2015).

The phylogenetic analysis of the major capsid protein was compared with other phages in the NCBI database using Mega-X software (version 10.1.6.). The inferred amino acid sequences’ alignment was performed with ClustalX using default parameters, followed by manually refining the alignments with Geneious (version 2020.1.2). The maximum likelihood tree was constructed with RAxML rapid bootstrapping (100 replicates) and the Jones-Taylor-Thornton model.

Genome comparison of PhiMa05 was analyzed using EasyFig version 2.2.3 (Sullivan et al., 2011). Multiple genome alignment for analysis of the genomic synteny was performed using the progressiveMauve (Darling et al., 2010) plugin in Geneious software (version 2020.1.2). A full list of phage genomes can be found in a Supplementary Table 2.

### Statistical Analysis

Data obtained were analyzed by ANOVA with the post hoc Tukey test using the SPSS statistical software version 17.0 for Windows EDU in order to investigate the significance of MCs production among different treatments of killing assay of *Microcystis* planktonic cells and aggregated cells at *P* ≤ 0.05.

## Results and Discussion

### Cyanophage Isolation and Plaque Forming Ability

**FIGURE 1:**
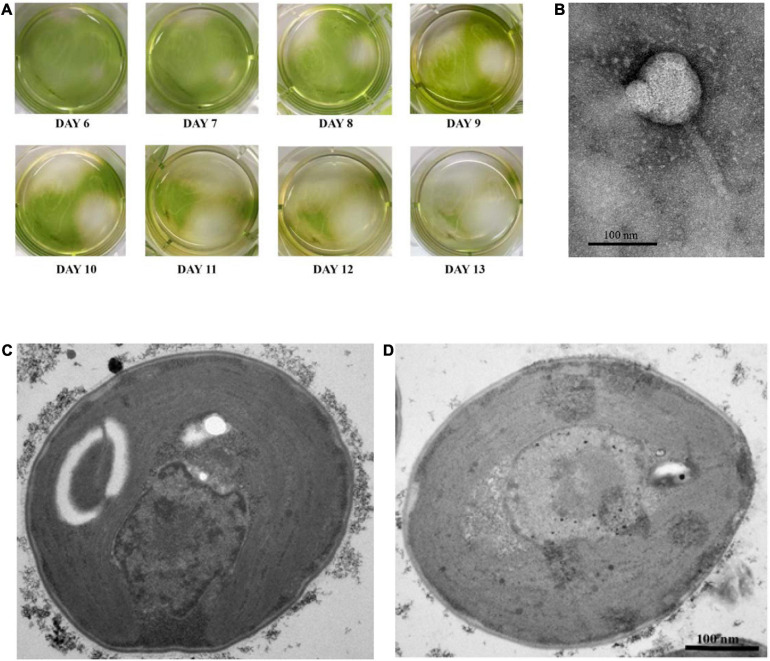
**(A)** Plaques of cyanophage PhiMa05 on the lawn of SG03. TEM images of: **(B)** Cyanophage PhiMa05; **(C)** thin section of a healthy cell of *Microsystis* SG03; and **(D)** thin section of *Microsystis* SG03 2 days after incubation with PhiMa05.

The wastewater samples were collected to test the presence of cyanophage against MCs-producing *Microcystis* SG03. This strain, previously confirmed by 16s RNA sequences (accession number MT534586) synthesized the highest IMCs contents (3.19 pg/cell) among our harmful *Microcystis* collection. Since the wastewater environment provides complexity of microbial ecosystem, it has been used to isolate phage for multiple applications, including phages against *Klebsiella pneumoniae* and *Escherichia coli* as potential therapeutic and biocontrol agents (Olsen et al., 2020; Wintachai et al., 2020). Based on the clearance of liquid culture, cyanophage designated PhiMa05 was identified with an ability to lyse SG03 within 3 days after infection. The plaque assay displayed transparent and round plaques with a diameter of approximately 4 mm on the lawns of SG03 culture on day 6 (Figure 1A). The plaque size increased daily to approximately 20 mm in diameter on day 13. Our plaque assay method was modified by using 0.5% low melted agarose instead of 0.75% conventional agar to overcome the burden of plaque forming. The less agarose content attributed phage diffusion to the medium, promoting phage propagation (Yuan and Gao, 2017; Wang et al., 2019). Because of the difficulty of plaque forming on 0.75% top agar, PhiMa05 was suspected to be a large sized virion, specifically a jumbo phage. Jumbo phages have been less isolated because they are often eliminated during the size-exclusion process of isolation, and it is hard for them to form plaque under the conventional method (Lewis et al., 2020). The liquid bioassay approach provided a shorter detection period due to the preference of cyanobacterial growth conditions in the water. Thus, this method is a suitable alternative jumbo-cyanophage isolation method comparable to the gold standard, double agar overlay assay.

### Phage PhiMa05 Morphology

A transmission electron microscopy (TEM) image of PhiMa05 revealed that virus particle exhibited tail phage with icosahedral head, a characteristic of the order *Caudovirales* (Figure 1B). The average length of the cyanophage from the top of the capsid to the end of the tail was 243 nm. The average icosahedral head width and height were 108 and 111 nm, respectively. The phage tail had a height of 127 nm and a width of 19 nm. With these morphological features, the PhiMa05 was characterized in the family of *Myoviriae*. Eight cyanophages specific to *Microcystis* sp. have been reported and belong to families: *Myoviridae* (2), *Podoviridae* (3), *Siphoviridae* (2), and Corticovirus-like particles (1) (Jaskulska and Mankiewicz-Boczek, 2020; Yang et al., 2020). Myoviruses exhibit an icosahedral capsid connected with a contractile tail, while siphoviruses have a long non-contractile tail, and podoviruses have a short non-contractile tail (Barylski et al., 2020). The corticovirus-like particles are tailless with an icosahedral capsid (Li et al., 2013). Two well-established myoviruses infecting *M. aeruginosa* are Ma-LMM01 and MaMV-DC isolated from a Japanese water reservoir and a Chinese freshwater lake (Yoshida et al., 2006; Ou et al., 2013). The heads of Ma-LMM01 and MaMV-DC had diameters of 86 and 70 nm, respectively, which are smaller than the PhiMa05 head. A healthy *Microcystis* cell is shown in Figure 1C in comparison with a thin section of the host cell after 2 days of incubation with PhiMa05 presented in Figure 1D, which revealed propagation of intracellular phage-like particles. This microscopic result suggests that PhiMa05 succeeded in entering hosts and propagation.

### Host Range and Phage Stability

**FIGURE 2:**
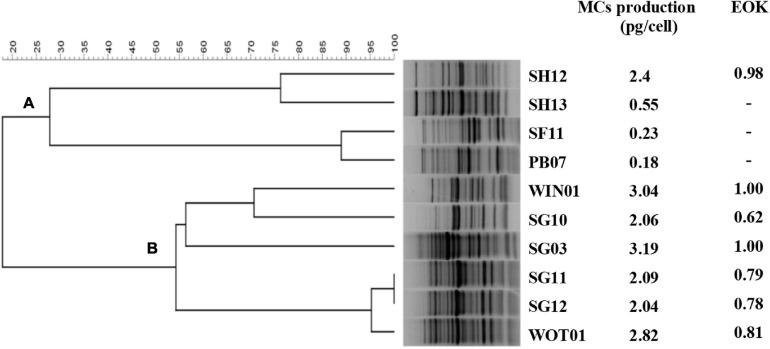
Dendrogram for *Microcystis* strains created by HIP PCR. HIP PCR was performed in 10 *Microcystis* isolates, creating 2 groups: **(A)** and **(B)**.

Host range and EOK were determined in 10 *Microcystis* strains. Both IMCs and EMCs productions after 5 days of inoculation were measured and only IMCs but not EMCs were detected in all strains (Figure 2). The clonal relatedness among the 10 isolates was investigated by HIP-PCR (Wilson et al., 2005), which has helped distinguish *Microcystis* genotypes (Jaiswal et al., 2013). The fingerprint patterns demonstrated high genetic variation. Two groups, A (*n* = 4) and B (*n* = 6), can be established (Figure 2). Members of group A showed less MCs production except for SH12. Cyanophage PhiMa05 can inhibit all group B members and the SH12. The result implies sharing receptors among these strains.

The EOK results agreed with the host range analysis. EOKs ranging from 0.62 to 1.00 were present after inoculating all group B members and SH12 with PhiMa05. This result indicates that the PhiMa05 infected toxic strains. Studies of host-specificity on freshwater cyanophage displayed that most of them are strain-specific, resulting from using only one host enrichment in the isolation process. Ma-LMM01 is a toxic strain-specific, whereas MaMV-DC is a non-toxic strain-specific (Yoshida et al., 2006; Ou et al., 2013). Our result indicates that PhiMa05 was also a narrow host range with toxin strain-specific rather than species-specific. The presence of these cyanophages in environments may affect the dynamic of toxic and non-toxic populations in cyanobacterial blooms.

As an alternative biological control agent, phage stability was determined under several stress conditions, including salinity, pH and temperature. PhiMa05 can withstand a wide range of salinity from 0.5 to 20 ppt after 12 and 24 h of incubations (Supplementary Figure 1A). The cyanophage viability decreased significantly when the concentrations of sodium chloride were at 30 and 40 ppt. PhiMa05 can also tolerate a wide range of pH. However, extreme pH conditions, pH 5 and 11, caused a significant decrease in the cyanophage viability (Supplementary Figure 1B). Additionally, the cyanophage PhiMa05 showed stability from 4°C to 35°C after 12 h and 24 h incubations. However, after exposure to 45°C for 24 h, there was a 23% reduction in phage viability (Supplementary Figure 1C). Our study is the first report of environmental stress on the stability of cyanophage. These tolerance characteristics suggest the strength of PhiMa05 in various environments. They could be an alternative biological method for inhibiting the growth of MCs-producing *Microcystis* and reducing the MCs accumulation in an aquatic environment.

### One Step Growth Curve

**FIGURE 3:**
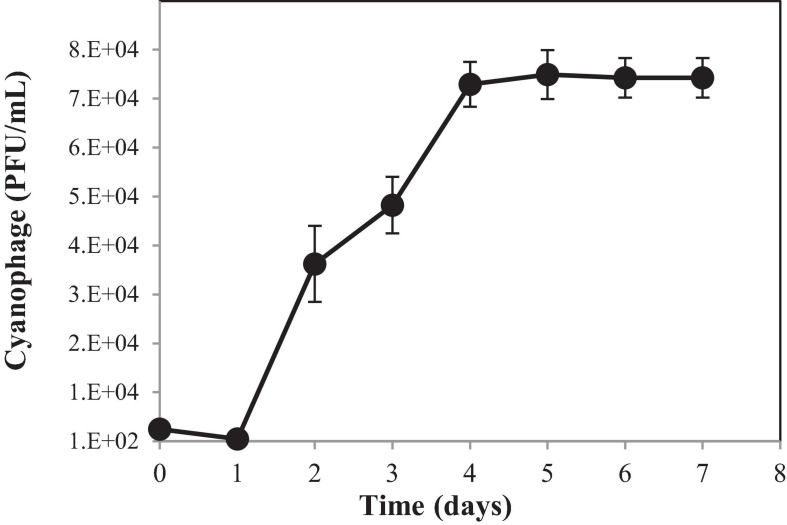
One-step growth curve of cyanophage PhiMa05. Data are the mean of triplicate independent experiments with standard deviation.

To understand the growth kinetics of PhiMa05, the one-step growth curve experiment was performed with the cyanophage at the MOI of 0.01 (Figure 3). The cyanophage latent period was estimated to be 1 day followed by a log period of approximately 3 days. The burst size of PhiMa05 was around 127 phage particles/infected cell. A total lysis was observed 4 days after inoculation. Up to date, only two studies have reported one step growth curve for cyanophage. Ou et al. (2013) reported *Microcystis* phage MaMV-DC with 2 days of latent period and a burst size of approximately 80 phage particles/infected cell. Another *Microcystis* phage, Ma-LMM01, revealed a faster latent period (12 h) with a burst size of 120 progeny virions without complete host cell lysis (Yoshida et al., 2006).

### Infection Dynamics in Planktonic Culture and Lysis-Mediated MCs Release

**FIGURE 4:**
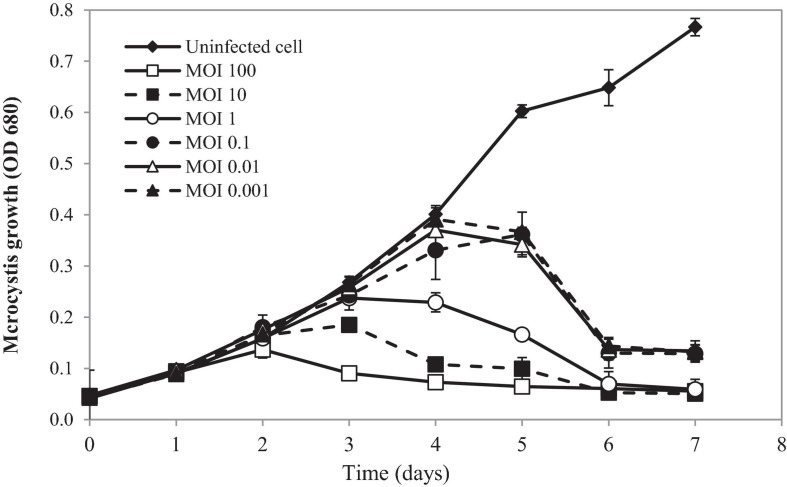
Time killing assay of cyanophage PhiMa05 against *Microcystis* SG03. The bacterial cultures were infected with the cyanophage at MOIs of 0.001, 0.01, 0.1, 1, 10, and 100. Uninfected bacterial culture was used as a control. Independent experiments were conducted in triplicate and optical density at 680 nm was measured, averaged, and plotted. Error bars are standard deviations.

Lytic activity of PhiMa05 at various MOIs (0.001–100) was demonstrated in Figure 4. Cyanophage PhiMa05 inhibited the growth of *Microcystis* SG03 in a concentration-dependent manner. The higher PhiMa05 concentration resulted in a faster decrease in SG03 number. At the MOIs of 1, 10 and 100, PhiMa05 effectively inhibited the SG03 growth. At the MOI of 100, the bacterial host (SG03) density decreased dramatically after 2 days of incubation, and then the bacterial population entirely collapsed within 3 days. The bacterial host reduction at the MOIs of 10 and 1 was observed after 3 and 4 days of incubation, respectively. At lower MOIs (0.001, 0.01, and 0.1), *Microcystis* growth was significantly inhibited after 5 days of inoculation. Phage titers were determined 7 days after inoculation. The highest phage densities (8 × 10^18^ infectious unit/ml) were obtained from infection at the MOIs of 0.001-1 (Supplementary Figure 2). These MOIs were optimal for phage propagation.

Our results suggest that using a higher phage ratio was suitable for phage therapy. The lower MOIs may provide additional time for phage infection resulting in host adaptation and cope phage infection (Watkins et al., 2014). However, Morimoto et al. (2018) revealed that Ma-LMM01 infecting *Microcystis* does not induce the antiviral defense response during the phage amplification.

**FIGURE 5:**
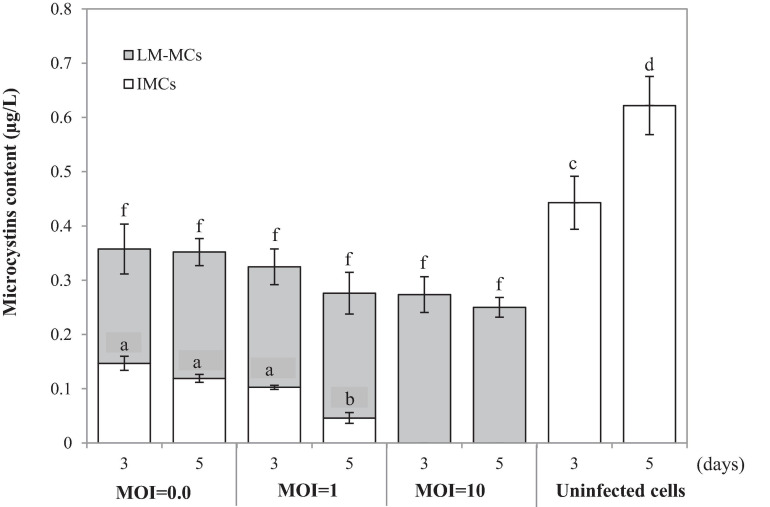
Changes in accumulated microcystins (IMCs) and lysis-mediated microcystins (LM-MCs) on days 3 and 5 of the control and PhiMa05 infected culture of *Microcystis* SG03 at MOIs of 0.01, 1, and 100. Each data point is the mean from three independent experiments. Error bars show standard deviations. Different letters above bars show values that are significantly different (*p* < 0.05). Each value is compared within each category.

To monitor the MCs after PhiMa05 infection at the MOIs of 0.01, 1, and 100, IMCs and LM-MCs were investigated after 3 and 5 days of incubation in comparison with the control, uninfected culture (Figure 5). The IMCs in the SG03 inoculum was 0.17 μg/L (day 0). In the control culture, MCs production significantly increased to 0.44 μg/L and 0.62 μg/L after 3 days and 5 days of incubation, respectively (*p* < 0.05). Infecting SG03 with PhiMa05 at all MOIs led to a significant MCs reduction (Figure 5). Phage PhiMa05 inhibited MCs production in a concentration-dependent manner, agreeing with the bacterial growth inhibition results. At the MOI of 100, PhiMa05 presumably infected all host cells within a short latent period (1 day). Then, SG03 host cells were entirely lysed after 3 days of inoculation, releasing MCs (LM-MCs) in the culture. Therefore, only LM-MCs but not IMCs and the host cells were detected. The LM-MCs of infected cells on days 3 and 5 were equal but were significantly less than IMCs from the control (*p* < 0.05). The incomplete killing was observed when the host was infected with phages at lower MOIs. At the MOIs of 0.01 and 1, the IMCs production significantly decreased after 3 days of infection, whereas the bacterial growth reduction was not observed (Figure 4). The total MCs productions were also reduced, implying that phages interfered with MCs production. The LM-MCs in the culture were detected after 3 and 5 days of infection, indicating the death of infected cells. Our results demonstrate that the cyanophage adversely affected the bacterial host population and suggest its functions in creating ecosystems’ stability.

### PhiMa05 Inhibitions of MCs Production and Aggregated Cells

**FIGURE 6:**
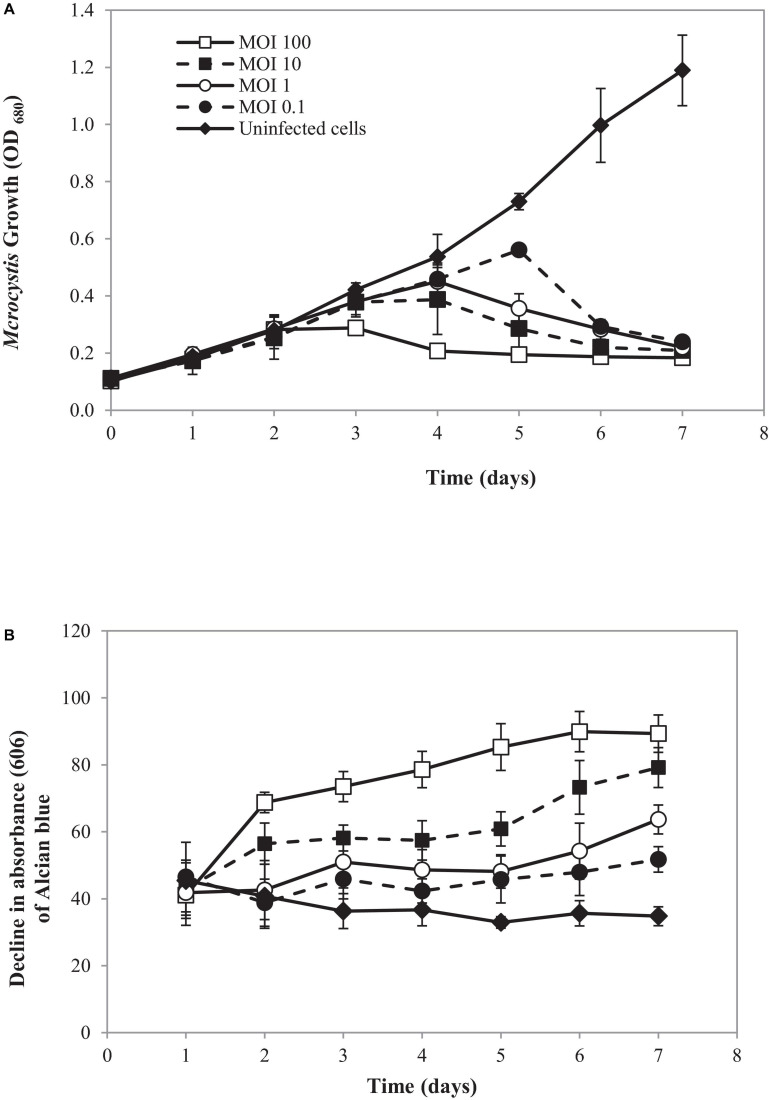
Effect of cyanophage PhiMa05 on aggregated *Microcystis* SG03. The aggregated cells were infected with the cyanophage at MOIs of 0.1, 1, 10, and 100. **(A)** Time-kill assay of cyanophage PhiMa05 against aggregated *Microcystis* SG03. **(B)** Effect of phage on bacterial aggregation. The data show the means ± standard deviations based on triplicated experiments.

In the natural ecosystem, divalent cations play a major role in *Microcystis* aggregation during blooming (Xu et al., 2016). The aggregation prolongs *Microcystis* in water bodies and enhances buoyancy (Drugǎ et al., 2019). At a laboratory scale, an aggregated form of SG03 was initiated by calcium addition (Drugǎ et al., 2019; Wei et al., 2019; Gu et al., 2020). The aggregated cells were infected by cyanophage PhiMa05 at different MOIs of 0.1, 1, 10, and 100. The density and cell-bound exopolymers of SG03 were determined daily within 7 days of infection. As shown in Figure 6, the aggregated SG03 cells were degraded by PhiMa05 in a concentration- and time-dependent manner. Reduction in cell density was seen after 3 days of infection. Within 7 days of infection, the aggregated cells were undetectable, indicating a total population collapse. At the MOI of 100, the bacterial density dramatically declined after 3 days of infection, and SG03 was entirely killed after 4 days of infection (Figure 6A).

Several studies have shown the involvement of exopolymer in cyanobacterial aggregated colonies (Boudarel et al., 2018; Liu et al., 2018; Bhatnagar and Bhatnagar, 2019). Here, exopolymer contents were determined through cell-bound exopolymers by measuring Alcian blue absorbed by aggregated cells (Vandevivere and Kirchman, 1993). After 2 days of infection at the MOIs of 10 and 100, bacterial clumps disaggregated, releasing dye (Alcian blue) residual to the culture. The disaggregation of bacterial clump was apparent after 3 days of infection with the MOIs of 1 and 0.1 (Figure 6B).

**FIGURE 7:**
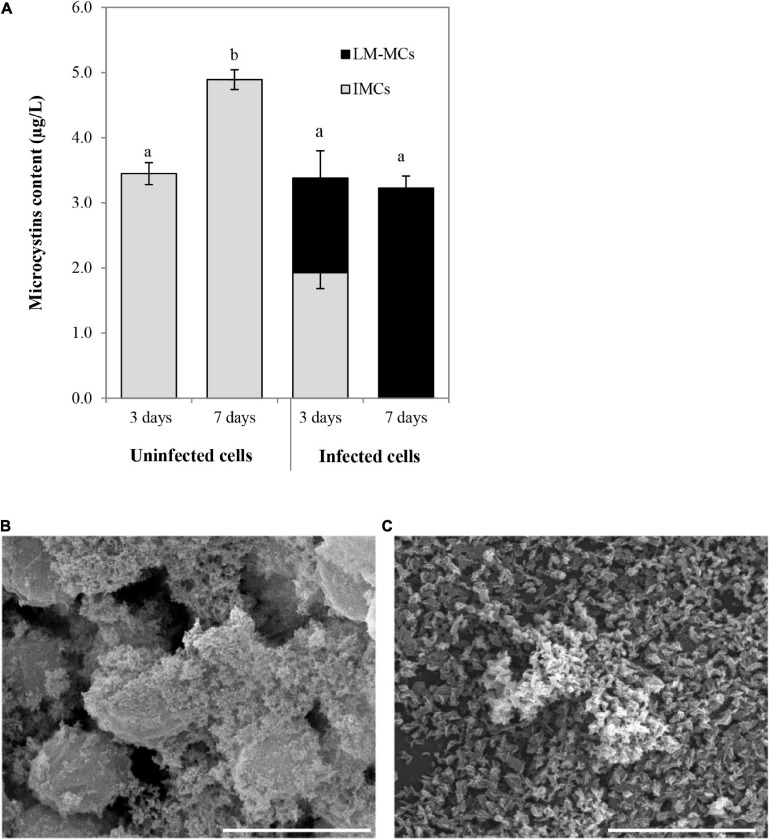
**(A)** Changes in accumulated microcystins (IMCs) and lysis-mediated microcystins (LM-MCs) after PhiMa05 infecting aggregated *Microcystis* SG03 at MOI of 100 on days 3 and 7. Each data point is the mean value from three independent experiments ± standard deviation (*P* value < 0.05). **(B,C)** Scanning electron micrographs after the phage treatment at MOI of 100: **(B)** Uninfected bacterial aggregation, **(C)** PhiMa05 infecting aggregated cells. Scale bar = 5 μm.

The MCs productions were determined in uninfected aggregated cells and bacterial aggregated colonies after cyanophage inoculation at the MOI of 100 (Figure 7A). In the beginning, the MCs content in the uninfected SG03 cells was 0.805 μg/L. After 3 and 7 days of incubation, IMCs but not LM-MCs were detected. On the other hand, LM-MCs and fewer IMCs were present with infected aggregated colonies indicating cyanobacterial death. Within 7 days of infection, PhiMa05 killed the entire aggregated colonies releasing LM-MCs without IMCs detection. Scanning electron microscopy (SEM) images revealed disaggregation and cell debris after 3 days of inoculation (Figures 7B,C). Our result indicates that after PhiMa05 attachment, aggregated cells were initially disrupted and then killed. The killing ability of phages against aggregated bacterial cells, including biofilm, has been demonstrated in other organisms such as *K. pneumoniae* and *Pseudomonas aeruginosa* (Wu et al., 2019; Oliveira et al., 2020; Wintachai et al., 2020). Our study is the first report of cyanophage activity against aggregated MCs-producing *Microcystis*.

Toxic cyanobacterial blooms are comprised of planktonic and aggregated cells. Our result showed that PhiMa05 is a good candidate for controlling toxic *Microcystis*, since conventional treatment processes are inefficient. The practical application of cyanophage to eliminate harmful cyanobloom was suggested by using high concentration cyanophages in a capsule (Mathieu et al., 2019). The phage will be released and infect surrounding cells. The limitations of this procedure involve environmental factors, including water flow and weather. The opportunity to use this approach in the water supply is more promising. Addition of cyanophages *in situ* could control toxic *Microcystis* without any modification to treatment facilities.

### PhiMa05 Genome Features and Analysis

The cyanophage PhiMa05 contains a linear double-stranded DNA genome of 273,876 bp in length with a GC content of 54.2%. Three current *Microcystis* viral genomes databases are available, including Ma-LMM01, MaMV-DC and Mic1 (Yoshida et al., 2008; Ou et al., 2015; Yang et al., 2020). The Mic1 is grouped in *Siphoviridae*, while PhiMa05 shared morphological characteristics with Ma-LMM01 and MaMV-DC, members of *Myoviridae*. All of them contain genome sizes ranging from 92 to 169 kb, while the PhiMa05 genome is larger. PhiMa05 is the first jumbo myovirus infecting MC producing *Microcystis*. The annotation of the PhiMa05 sequence revealed the presence of 254 predicted ORFs. All the ORFs showed an ATG start codon. One hundred thirty-three ORFs were presented on the negative strand, with the remaining ORFs on the positive strand. Yuan and Gao (2017) demonstrated that jumbo phages have diverse origins and carry essential genes for the phage life cycle and extra genes obtained from their host during the evolutionary process (Yuan and Gao, 2017).

To search homologs of the PhiMa05 predicted proteins, ORFs were annotated with protein available in the NCBI database. Only 54 ORFs were identified as putative functional proteins, and 12 ORFs were assigned hypothetical proteins (Supplementary Table 1). A total of 16 known ORFs were homologous to protein from uncultured *Caudovirales* phage, and four ORFs show a degree of similarity to *Myoviridae* proteins (Supplementary Table 1). However, most of the putative proteins presented a lower percentage of similarity than other phages in the database. All predicted ORFs did not match with any proteins from the Ma-LMM01 and MaMV-DC. The genomes of cyanoviruses infecting freshwater cyanobacteria have displayed less homology to each other (Morimoto et al., 2020).

The putative functional genes of PhiMa05 were analyzed (Supplementary Table 1) and separated into 4 groups comprising AMGs, DNA replication and nucleotide metabolism, host cell lysis and structural proteins. The AMGs in cyanophage are derived from cyanobacterial hosts (Thompson A.W. et al., 2011). After infection, the host metabolism is shut off, and then phages boost and redirect host metabolism via AMGs (Thompson et al., 2016). The proteins encoded by AMGs are crucial for energy metabolism (photosynthesis, carbon metabolism, and phosphorus utilization), providing ATP or reducing power for nucleotide biosynthesis and phage genome replication (Thompson A.W. et al., 2011; Enav et al., 2014; Puxty et al., 2015). Here, PhiMa05 possesses ORF228-encoded S-adenosylmethionine decarboxylase (*SpeD*), a key enzyme for maintaining the host PSII reaction center’s activity during phage infection (Bograh et al., 1997; Sullivan et al., 2005). In addition, the photosynthetic electron transport as ferredoxin (ORF1) was identified in PhiMa05, as a function in redirection of the electron transport chain (Puxty et al., 2015) and so was the electron acceptor as ferredoxin-NADP reductase (ORF48) for the generation of NADPH during infection (Thompson et al., 2016). Several genes involving dNTP synthesis and energy production in the tricarboxylic acid cycle (TCA) have been reported in cyanophage genomes (Sullivan et al., 2010; Thompson L.R. et al., 2011). Here, phosphopantetheine adenylyltransferase (ORF34), isocitrate dehydrogenase (ORF56), and phosphoglucomutase (ORF204) involving in the TCA cycle were detected in PhiMa05. In contrast to most cyanophage, PhiMa05 lacked of carbon metabolic genes in the pentose phosphate pathway and the Calvin cycle. Only phosphoribosylpyrophosphate synthetase (ORF79) for dNTP biosynthesis existed (Hurwitz and U’Ren, 2016).

We identified 25 genes involved in DNA replication, recombination, and repair in PhiMa05 (Table 1). The nucleotide biosynthesis genes have been reported in T4-like cyanophage genomes, including ribonucleotide reductase (RNRs) (Hanson and Mathews, 1994; Gao et al., 2016). Lytic T4-like phages but not temperate phages present the RNRs (Chen and Lu, 2002). Here, the ORF58 of PhiMa05 was RNR-encoded genes, indicating that RNRs degrade the host DNA during cyanophage replication. The products of ORF60 and ORF208 were RNAP sigma subunits, which are essential inhibitors of the host RNAP (Bae et al., 2013). ORF11 was encoded for thioredoxin reductase (gp344), which has been predicted to be responsible for DNA metabolism and replication and structural proteins (Danis-Wlodarczyk et al., 2018). Morimoto et al. (2018) suggested that cyanophages use their genes in transcription and translation, keeping the host in a stable metabolic state. Therefore, host antiviral responses are less virulent, allowing cyanophage propagation.

**TABLE 1:** Predicted ORFs of cyanophage PhiMa05 with similarity to genes of know function.

ORF	Putative proteins	Phage	*E*-value	Identity (%)	GenBank ID
8	Tryptophanyl-tRNA synthetase	*Myoviridae* sp.	1.00E-20	30	AXH72876.1
11	Putative RNA polymerase sigma subunit	*Bacillus* phage AP631	5.00E-21	37	AZF88386.1
36	Transcription antitermination protein	*Sinorhizobium* phage phi3LM21	4.00E-14	28	ATE84713.1
58	Ribonucleoside-diphosphate reductase	*Loktanella* phage pCB2051-A	9.00E-10	35	YP_007674964.1
60	RNA polymerase sigma factor	*Klebsiella* phage ST147-VIM1phi7.1	4.00E-82	50	YP_009882596.1
76	DNA polymerase IV	*Paracoccus* phage vB_PthS_Pthi1	9.00E-24	30	AZV00415.1
94	PcnB tRNA nucleotidyltransferase/poly(A) polymerase	Uncultured *Caudovirales* phage	8.80E-12	24	CAB4197116.1
104	Pentapeptide repeat	Uncultured *Caudovirales* phage	5.00E-27	40	CAB5225639.1
106	GTP-binding protein	*Klebsiella* phage ST512-KPC3phi13.3	6.00E-06	26	QBQ71826.1
112	Glycine-tRNA ligase beta subunit	*Yersinia* phage vB_YpM_Tongde	4.00E-06	46	QMP18904.1
132	DNA repair exonuclease SbcCD ATPase subunit	Phage 5P_2	8.00E-06	35	AZF90183.1
136	GIY-YIG nuclease superfamily protein	Vibrio phage 1.031.O._10N.261.46.F8	2.00E-07	35	AUR82991.1
144	MutT-like nucleotide pyrophosphohydrolase	*Streptomyces* phage Bmoc	1.00E-04	28	QJD50776.1
148	TopA topoisomerase IA	Uncultured *Caudovirales* phage	6.00E-123	37	CAB4197034.1
177	Leucine-tRNA ligase	*Staphylococcus* phage UPMK_1	3.00E-04	22	ATW69266.1
183	PurB Adenylosuccinate lyase	Uncultured *Caudovirales* phage	2.00E-74	36	CAB4177963.1
188	Threonine-tRNA ligase	*Vibrio* phage Va_90-11-286_p16	9.00E-10	26	QCW19681.1
189	FusA translation elongation factors (GTPases)	Uncultured *Caudovirales* phage	5.00E-25	38	CAB4196865.1
208	RNA polymerase sigma-W factor	crAssphage cr118_1	8.00E-15	30	QOR58402.1
211	LysU Lysyl-tRNA synthetase (class II)	Uncultured *Caudovirales* phage	3.00E-75	25	CAB4196783.1
221	Amidophosphoribosyltransferase	*Wolbachia* phage WO	6.00E-105	42	QHJ75434.1
222	Putative phosphoribosyl formylglycinamidine (FGAM) synthase II	*Microbacterium* phage Min1	3.00E-173	40	YP_001294830.1
224	Pentapeptide repeat family protein	*Caulobacter* phage RW	2.00E-20	40	QDH50377.1
242	RNA binding protein	*Streptomyces* phage Abt2graduatex2	4.00E-10	28	ATN93725.1
248	MerR family transcriptional regulator	*Streptococcus* phage Javan105	4.00E-07	32	QBX13757.1

We detected 2 genes (ORF134 and 191) encoding for peptidases involving host cell lysis in PhiMa05. The putative Zn-dependent peptidase (ORF134) was reported to cleave *N*-acetylmuramic acid of peptidoglycan resulting in cell wall lysis (Hermoso et al., 2007). Another protein, peptidase M15 (ORF191) was proposed as putative endolysins among *Myoviridae* members (Walmagh et al., 2012; Antonova et al., 2019). None of predicted proteins involving lysogeny functions was found in the PhiMa05 genome, including integrase, transposase, excisionase, repressor, and genome attachment site (CI and CII proteins) (Lamont et al., 1993; Golais et al., 2013; Altamirano and Barr, 2019). This evidence suggested that PhiMa05 was a lytic phage.

**FIGURE 8:**
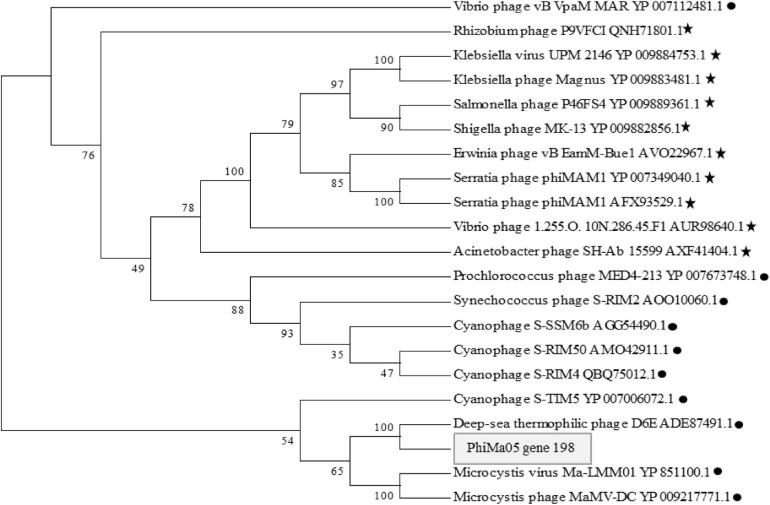
Maximum likelihood amino acid tree of the PhiMa05 major capsid protein. Boostraps values are shown (100 replicate). Black circle and star represent phage family of *Myoviridae* and *Ackermannviridae*, respectively.

**FIGURE 9:**
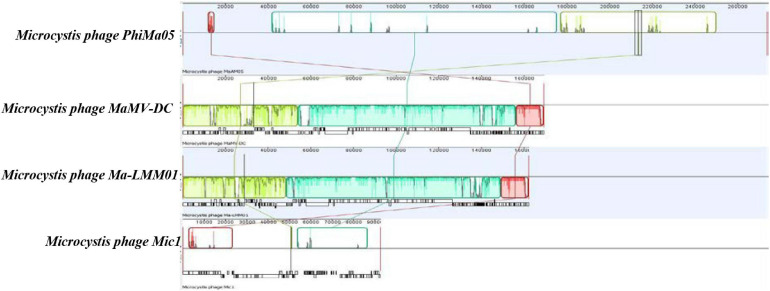
Comparative genome analysis of phage PhiMa05 genome with *Microcystis* phages. The colored collinear blocks indicate homologous regions between genome sequences while the height of the similarity profile in the collinear blocks indicates average level of conservation in the regions of the genome sequence.

ORF 198, 119, and 201 in PhiMa05 showed the best hits with structural proteins; capsid protein, and portal protein, respectively. An evolutionary analysis in bacteriophages has been based on the capsid protein and terminase large subunit sequence. Since the terminase was not found in the PhiMa05 genome, a phylogenetic tree based on the capsid sequence was generated (Figure 8). This protein sequence of PhiMa05 was closely related to deep-sea thermophilic bacteriophage D6E (40% sequence identity with 99% query coverage), unclassified *Myoviridae* (Figure 8), suggesting that the relationships among marine and freshwater cyanoviruses. This evidence agrees with Morimoto et al. (2020) that the cyanoviruses co-evolved independently with the host origin. Genome comparative analysis with other phages in the database indicated that PhiMa05 is unique as it shares ANI < 1%. Therefore, PhiMa05 is a new phage species. Genome alignment PhiMa05 with *Microcystis* cyanophages (Ma-LMM01, MaMV-DC, and Mic1) was shown in Figure 9. Three collinear blocks were shared among PhiMa05, Ma-LMM01 and MaMV-DC with different arrangements and lengths. No synteny and very low sequence similarities were observed. Alignment with four jumbo phages revealed no shared collinear blocks and sequence similarities (Supplementary Figure 3). Interestingly, *g91* encoding the sheath protein of the contractile tail was not detected in the PhiMa05 genome. The *g91* has been proposed to be conserved among myovirus infecting *M. aeruginosa* (Jaskulska and Mankiewicz-Boczek, 2020). This finding indicates the evolutionary divergence of PhiMa05 apart from other reported phages.

## Conclusion

Although viral infections have been considered a promising strategy for controlling harmful cyanobacterial populations, dynamics of toxin-release infection by cyanophage have not been reported. This study demonstrates the characteristics of myovirus PhiMa05 specific to MCs-producing *Microcystis* and its genome. The PhiMa05 lysed toxin-producing strains of *Microcystis*, with a large burst size, rapid killing, and high stability under various environmental stresses. Based on the infection experiments, lysis-mediated MCs were detected. PhiMa05 could kill both planktonic and aggregated cells and interfere with MCs productions. Applications of using PhiMa05 as a bio-control should be further investigated. The whole-genome sequence analysis suggests that PhiMa05 was a jumbo phage possessing several genes, including AMGs, structural proteins and other genes involving in phage DNA replication and host cell lysis. Based on the plaque formation assay and genome analysis, PhiMa05 was the virulent phage without any genes correlated to lysogenic phage, toxin, or antibiotic resistance. However, future studies are needed to explore (1) the transcriptional program of PhiMa05-host during infection and ecological roles, and (2) the safety of potential PhiMa05 applications because most of ORFs are putative and their functions are unknown.

## Data Availability Statement

The datasets presented in this study can be found in online repositories. The names of the repository/repositories and accession number(s) can be found below: NCBI, MW495066.1.

## Author Contributions

RP, OS, and AN contributed to the experimental design. AN performed the experiment and drafted the manuscript. KS and AN contributed to the bioinformatics analysis. RP contributed to the data analysis, manuscript discussion, and revision. EK and RP edited and approved the final content manuscript. All authors contributed to the article and approved the submitted version.

## Conflict of Interest

The authors declare that the research was conducted in the absence of any commercial or financial relationships that could be construed as a potential conflict of interest.
