# Supplementary material for: A Novel Jumbo Phage PhiMa05 Inhibits Harmful Microcystis sp
Source: Front Microbiol. 2021 Apr 20;12:660351. doi: 10.3389/fmicb.2021.660351 (PMC8093824; doi:10.3389/fmicb.2021.660351)
Supplement: Supplementary file 1 [file Data_Sheet_1.PDF]

Supplementary Material

(A)

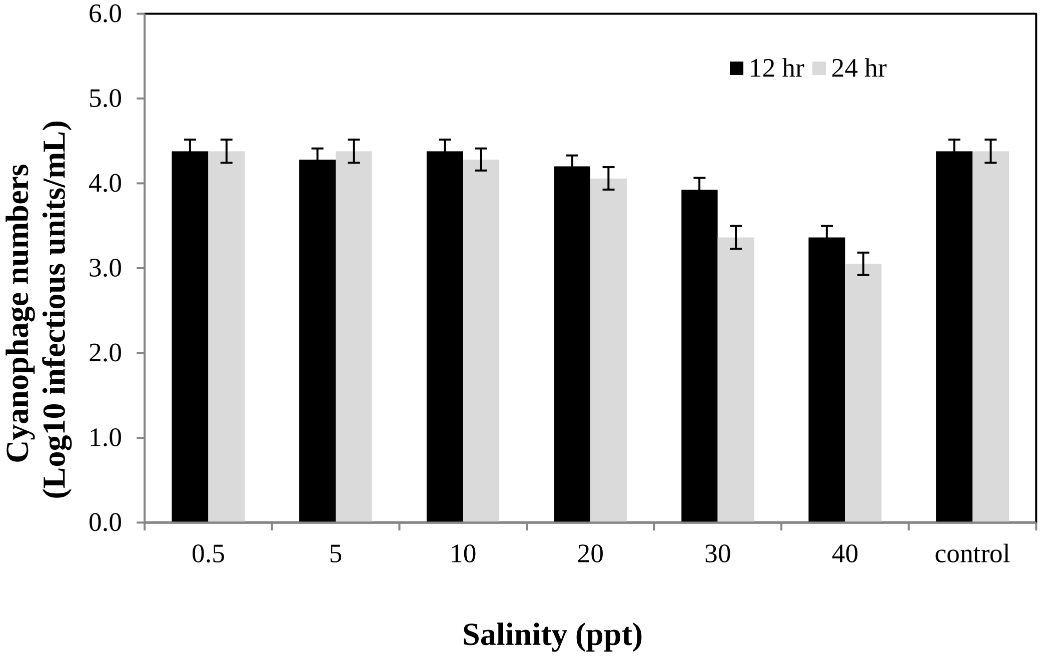

(B)

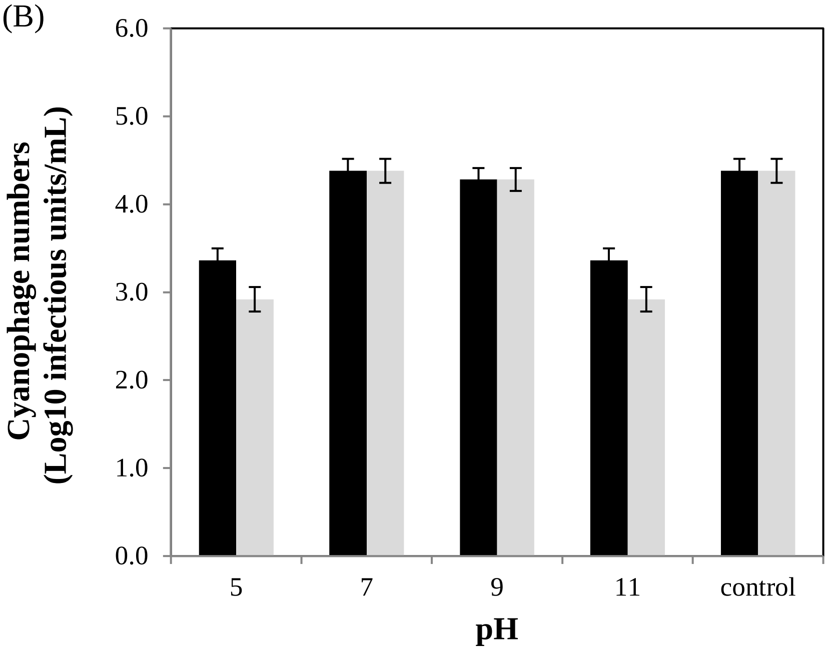

(C)

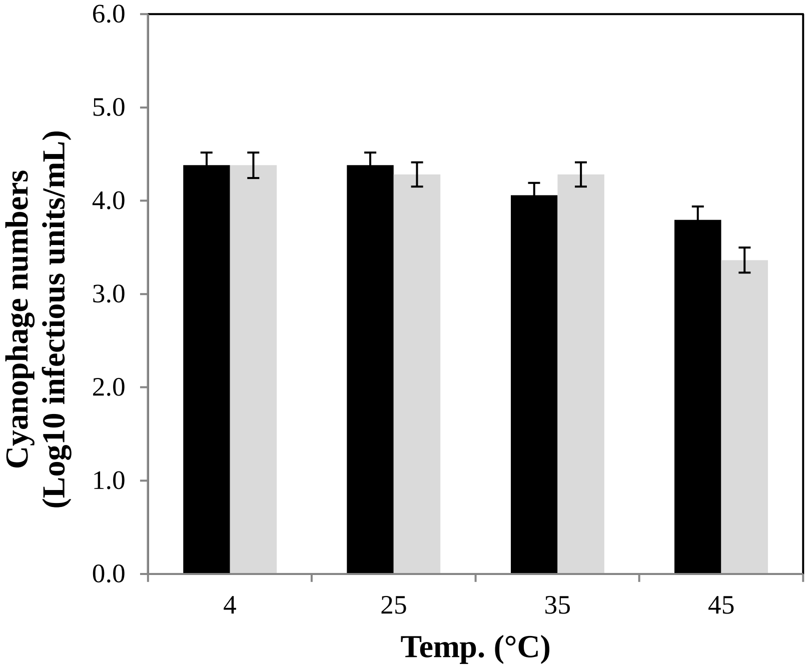

Figure 1S phage stability under different conditions. (A) Salinity, (B) pH, and (C) Temperature.

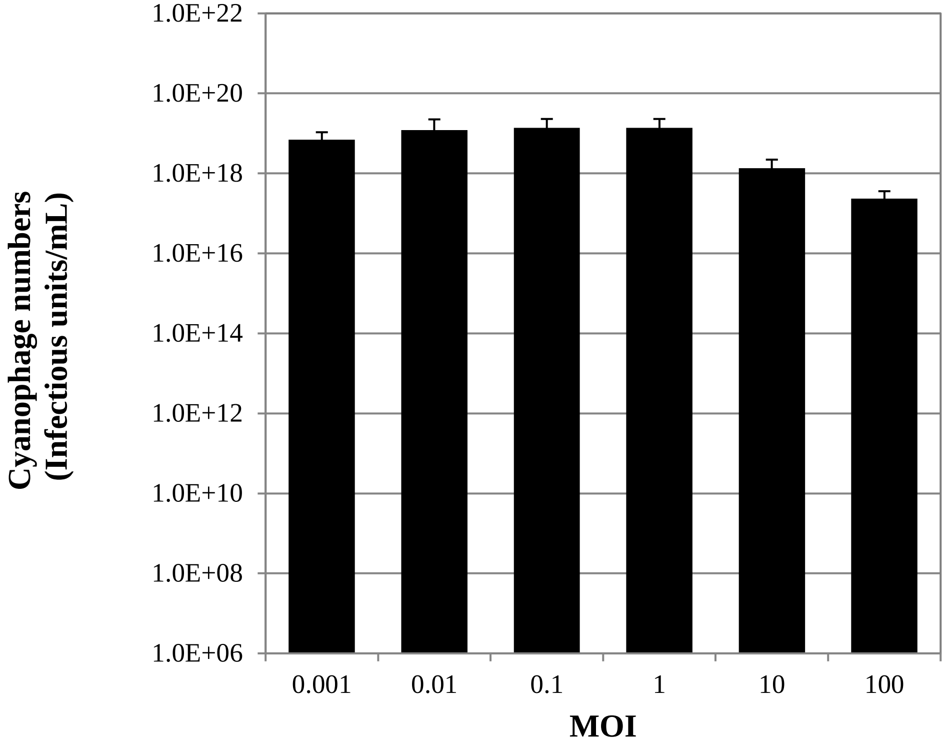

Figure 2S The cyanophage (PhiMa05) numbers on day 7 after *Microcystis* SG03 infected at the MOIs of 0.001, 0.01, 0.1, 1, 10, and 100.

Table 1S Predicted ORFs of cyanophage PhiMa05with genes of known functions.

ORF	Putative proteins	Phage	Query coverage	E-value	Identity (%)	GenBank ID
1	4-hydroxy-3-methylbut-2-enyl-diphosphate synthase	Klebsiella phage ST512-KPC3phi13.3	92	1.00E-107	45	QBQ71814.1
8	tryptophanyl-tRNA synthetase	Myoviridae sp.	61	1.00E-20	30	AXH72876.1
11	putative RNA polymerase sigma subunit	Bacillus phage AP631	91	5.00E-21	37	AZF88386.1
20	aerotaxis sensor receptor protein	Escherichia virus P1	38	2.00E-20	30	AXN57531.1
23	CpaF Flp pilus assembly protein, ATPase CpaF	uncultured Caudovirales phage	66	3.00E-28	30	CAB4192406.1
29	thioredoxin reductase gp344	Bacillus virus G	97	3.00E-76	42	YP_009015647.1
34	CoaD Phosphopantetheine adenylyltransferase	uncultured Caudovirales phage	92	7.00E-30	39	CAB4196827.1
36	transcription antitermination protein	Sinorhizobium phage phi3LM21	75	4.00E-14	28	ATE84713.1
39	AhpC Peroxiredoxin	uncultured Caudovirales phage	95	1.00E-24	30	CAB4137972.1
45	unnamed protein product	[Planktothrix phage PaV-LD]	66	3.00E-09	50	YP_004957389.1
48	ferredoxin/ferredoxin--NADP reductase	[uncultured Mediterranean phage uvMED]	95	5.00E-16	46	BAQ85388.1
50	sensor domain-containing diguanylate cyclase	Klebsiella phage ST846-OXA48phi9.1	49	3.00E-17	28	QBP07796.1
56	isocitrate dehydrogenase, partial	Phage 21	13	1.00E-11	50	AAC48877.1
58	ribonucleoside-diphosphate reductase	Loktanella phage pCB2051-A	93	9.00E-10	35	YP_007674964.1
60	RNA polymerase sigma factor	Klebsiella phage ST147-VIM1phi7.1	75	4.00E-82	50	YP_009882596.1
62	pentapeptide	Rheinheimera phage vB_RspM_Barba5S	44	1.00E-07	24	YP_009822637.1
71	bifunctional NAD-dependent-3-hydroxypropionate dehydrogenase	Klebsiella phage ST13-OXA48phi12.4	93	6.00E-24	30	QBQ71967.1
75	Protein of unknown function DUF3228	uncultured Caudovirales phage	96	9.00E-35	36	CAB4196785.1
76	DNA polymerase IV	Paracoccus phage vB_PthS_Pthi1	58	9.00E-24	30	AZV00415.1
79	PrsA Phosphoribosylpyrophosphate synthetase	uncultured Caudovirales phage	92	9.00E-102	48	CAB4123205.1

Table 1S Predicted ORFs of cyanophage PhiMa05 with genes of known function. (Cont.)

ORF	Putative proteins	Phage	Query coverage	E-value	Identity (%)	GenBank ID
82	hypothetical protein	Acinetobacter phage Ab105-3phi	89	1.00E-06	32	ALJ98965.1
92	3-polyprenyl-4-hydroxybenzoate carboxy-lyase	Wolbachia phage WO	90	1.00E-37	39	QHJ75468.1
94	PcnB tRNA nucleotidyltransferase/poly(A) polymerase	uncultured Caudovirales phage	71	8.80E-12	24	CAB4197116.1
97	proline dehydrogenase	Staphylococcus phage UPMK_1	85	5.00E-43	31	ATW69272.1
100	hypothetical protein	Myoviridae sp.	27	4.00E-06	38	AXH72863.1
104	Pentapeptide repeat	uncultured Caudovirales phage	98	5.00E-27	40	CAB5225639.1
105	enoyl-[acyl-carrier-protein] reductase [NADH]	Klebsiella phage ST11-VIM1phi8.2	90	9.00E-81	49	QBP28530.1
106	GTP-binding protein	Klebsiella phage ST512-KPC3phi13.3	87	6.00E-06	26	QBQ71826.1
111	penicillin-insensitive transglycosylase & transpeptidase	Klebsiella phage ST512-KPC3phi13.3	78	8.00E-45	30	QBQ71818.1
112	glycine--tRNA ligase beta subunit	Yersinia phage vB_YpM_Tongde	9	4.00E-06	46	QMP18904.1
119	tail fibers protein	Escherichia phage vB_EcoM_PHB05	19	4.90E-03	32	ATI15877.1
132	DNA repair exonuclease SbcCD ATPase subunit	Phage 5P_2	6	8.00E-06	35	AZF90183.1
133	gp2	Bacillus virus G	88	2.00E-22	24	YP_009015313.1
134	putative Zn-dependent peptidase	Myoviridae sp.	69	2.00E-09	20	AXH72868.1
136	GIY-YIG nuclease superfamily protein	Vibrio phage 1.031.O._10N.261.46.F8	86	2.00E-07	35	AUR82991.1
144	MutT-like nucleotide pyrophosphohydrolase	Streptomyces phage Bmoc	81	1.00E-04	28	QJD50776.1
148	TopA Topoisomerase IA	uncultured Caudovirales phage	76	6.00E-123	37	CAB4197034.1
172	aldehyde dehydrogenase B	Pseudomonas phage vB_Pae_BR58b	33	4.00E-04	26	QBI82341.1
177	leucine-tRNA ligase	Staphylococcus phage UPMK_1	47	3.00E-04	22	ATW69266.1
183	PurB Adenylosuccinate lyase	uncultured Caudovirales phage	86	2.00E-74	36	CAB4177963.1
187	cell division protease	Myoviridae sp.	69	3.00E-158	47	QMP83380.1
188	threonine-tRNA ligase	Vibrio phage Va_90-11-286_p16	22	9.00E-10	26	QCW19681.1
189	FusA Translation elongation factors (GTPases)	uncultured Caudovirales phage	61	5.00E-25	38	CAB4196865.1
191	peptidase M15	Nonlabens phage P12024S	93	2.00E-25	41	YP_006560353.1
194	hypothetical protein UFOVP93_7	uncultured Caudovirales phage	97	1.00E-05	31	CAB4127669.1
195	hypothetical protein	uncultured Mediterranean phage uvMED	98	1.00E-11	20	BAR35929.1

Table 1S Predicted ORFs of cyanophage PhiMa05 with genes of known function. (Cont.)

ORF	Putative proteins	Phage	Query coverage	E-value	Identity (%)	GenBank ID
196	hypothetical protein UFOVP356_26	uncultured Caudovirales phage	94	8.00E-07	25	CAB4139932.1
198	capsid protein	Deep-sea thermophilic phage D6E	99	5.00E-63	40	YP_007010926.1
201	portal protein	Cyanophage PP	96	3.00E-71	31	YP_008766993.1
204	Phosphoglucomutase	Streptococcus phage phiJH1301-2	87	1.00E-27	25	ANM47632.1
208	RNA polymerase sigma-W factor	crAssphage cr118_1	90	8.00E-15	30	QOR58402.1
211	LysU Lysyl-tRNA synthetase (class II)	uncultured Caudovirales phage	57	3.00E-75	25	CAB4196783.1
212	BaeS Signal transduction histidine kinase	uncultured Caudovirales phage	34	2.00E-13	32	CAB4129709.1
221	amidophosphoribosyltransferase	Wolbachia phage WO	95	6.00E-105	42	QHJ75434.1
222	putative phosphoribosyl formylglycinamidine (FGAM) synthase II	Microbacterium phage Min1	97	3.00E-173	40	YP_001294830.1
224	pentapeptide repeat family protein	Caulobacter phage RW	74	2.00E-20	40	QDH50377.1
226	SpeB Arginase/agmatinase/formimionoglutamate hydrolase, arginase family	uncultured Caudovirales phage	89	4.00E-35	32	CAB4123030.1
227	SpeE Spermidine synthase	uncultured Caudovirales phage	85	2.00E-61	40	CAB4129971.1
228	SpeD S-adenosylmethionine decarboxylase	uncultured Caudovirales phage	85	1.00E-15	34	CAB4124939.1
231	bactoprenol glucosyl transferase	Cronobacter phage ENT47670	68	4.00E-14	30	YP_007237578.1
235	ATP-dependent protease gp262	Bacillus virus G	54	2.00E-26	42	YP_009015565.1
242	RNA binding protein	Streptomyces phage Abt2graduatex2	42	4.00E-10	28	ATN93725.1
243	hypothetical protein	Streptococcus phage MM1	53	9.00E-58	40	CAB96620.1
248	MerR family transcriptional regulator	Streptococcus phage Javan105	46	4.00E-07	32	QBX13757.1
249	iron-sulfur cluster assembly protein	Staphylococcus phage phiSP44-1	96	6.00E-142	46	AZB66618.1
254	hypothetical protein SEA_PEPE_81	Mycobacterium phage Pepe	91	4.00E-12	33	YP_009189952.1

Table 2S A full list of genome phages, including phage PhiMa05 (bold)

	Phage name	Classification	Accession	Genome length (bp)
1	*Microcystis* phage Mic1	Siphoviridae	MN013189.1	92627
2	*Microcystis* phage MaMV-DC	Myoviridae	KF356199.1	169223
3	*Microcystis* phage Ma-LMM01	Myoviridae	AB231700.1	162109
4	*Ralstonia* phage RP12	Myoviridae	AP017924.1	279845
5	*Vibrio* phage BONAISHI	Myoviridae	MH595538.1	288967
6	*Xanthomonas* phage Xoo-sp14	Myoviridae	MT939492.1	232104
7	*Pseudomonas* phage vB_PaeM_MIJ3	Myoviridae	LR588166.1	288170
8	***Microcystis* phage PhiMa05**	Myoviridae	MW495066.1	273876

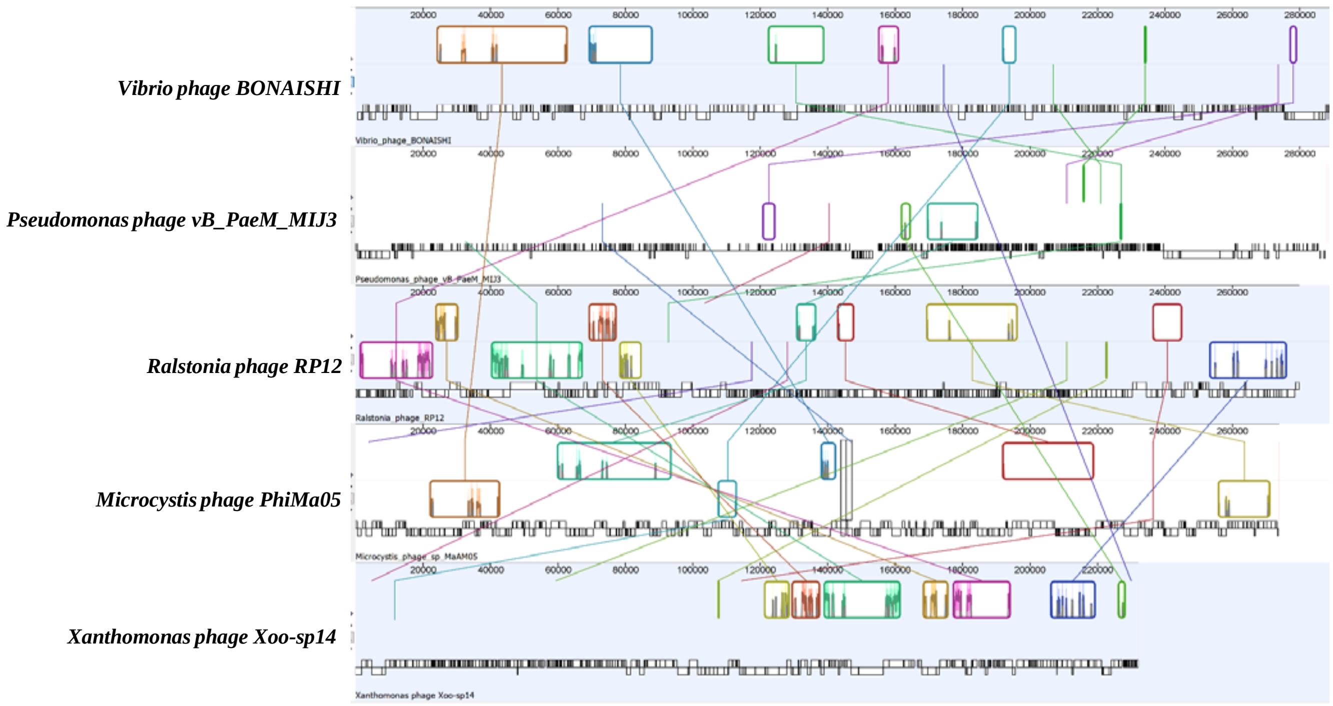

Figure 3S Comparative genome analysis of phage PhiMa05 genome with jumbo phages. The colored collinear blocks indicate homologous regions between genome sequences, while the height of the similarity profile in the collinear blocks indicates the average level of conservation in the regions of the genome sequence.
